# Deficits in growth, muscle mass, and body composition following placental insufficiency-induced intrauterine growth restriction persisted in lambs at 60 d of age but were improved by daily clenbuterol supplementation

**DOI:** 10.1093/tas/txaa097

**Published:** 2020-12-22

**Authors:** Rachel L Gibbs, Rebecca M Swanson, Joslyn K Beard, Ty B Schmidt, Jessica L Petersen, Dustin T Yates

**Affiliations:** Department of Animal Science, University of Nebraska–Lincoln, Lincoln, NE

## INTRODUCTION

Low birthweight in livestock results from stress-induced intrauterine growth restriction (IUGR; Yates et al., 2018). IUGR fetuses exhibit diminished muscle growth that persists in the neonatal stage, leading to asymmetric body composition and decreased weight gain (Cadaret et al., 2019). Ultimately, low birthweight diminishes yield and carcass merit at harvest (Greenwood et al., 2000), making effective postnatal treatment strategies to improve IUGR growth outcomes necessary. In this study, we examined the benefits of injecting the β2 agonist clenbuterol daily to target adrenergic adaptations that we previously observed in IUGR muscle (Posont et al., 2018; Yates et al., 2018). We hypothesized that IUGR-induced growth deficits would persist at the juvenile stage, manifesting in inferior body composition and carcass traits. We also postulated that clenbuterol would at least partially recover growth and body symmetry. Our objective was to test this hypothesis by assessing growth metrics and body composition in IUGR-born lambs hand-reared to 60 d of age and supplemented daily with injectable clenbuterol.

## MATERIALS AND METHODS

These studies were approved by the Institutional Animal Care and Use Committee at the University of Nebraska–Lincoln, which is accredited by AAALAC International. Placental insufficiency-induced IUGR lambs were produced from Polypay ewes as previously described (Cadaret et al., 2019). Briefly, timed-mated ewes were housed at 40 °C, 35% humidity from the 40th to 95th days of gestational age, then returned to 25 °C alongside their pair-fed controls until lambing. Lambs were weaned at 12 h of age, raised on milk replacer (Land O’Lakes) for the first 30 d, and transitioned to an ad libitum grain diet by 45 d of age. At birth, IUGR lambs were randomly assigned to receive clenbuterol (0.08 μg/kg in 1 mL saline; VetOne; *n* = 4) or saline only (*n* = 6). All control lambs (*n* = 7) received saline only. Bodyweights (BW), crown circumference, front cannon bone length, body girth, and crown-rump length were measured at birth, 30 d, and 60 d. Lambs were euthanized at 60 d of age and internal organs and flexor digitorum superficialis (FDS) muscles were weighed. Lamb carcasses were chilled for 24 h, and loin-eye area was measured between the 12th and 13th ribs. Bioelectrical impedance analysis was performed in live lambs at 30 and 60 d and on the loin muscle at necropsy as previously described (Gibbs et al., 2019). Briefly, a Quantum V (RJL Systems) was used to measure reactance, resistance, and phase angle from two sets of equally spaced electrodes connected to aluminum 20G MONOJECT needles placed subcutaneous (SQ) in live animals and intramuscular (IM) in the loin. Outer electrodes were 2.5 cm behind the point of the scapula and 5 cm in front of the hip bone, 1 cm off the midline. Inner electrodes were 2.5 cm inside of these. Six 30-s measurements were averaged. Proximate analysis was performed on the contralateral loin muscle (Midwest Labs). Data were analyzed by ANOVA using the mixed procedure of SAS. For growth metrics, day was treated as a repeated measure. Lamb was considered the experimental unit, and significant differences were declared at α ≤ 0.05 and tendencies at α ≤ 0.10.

## RESULTS

**Figure 1.**
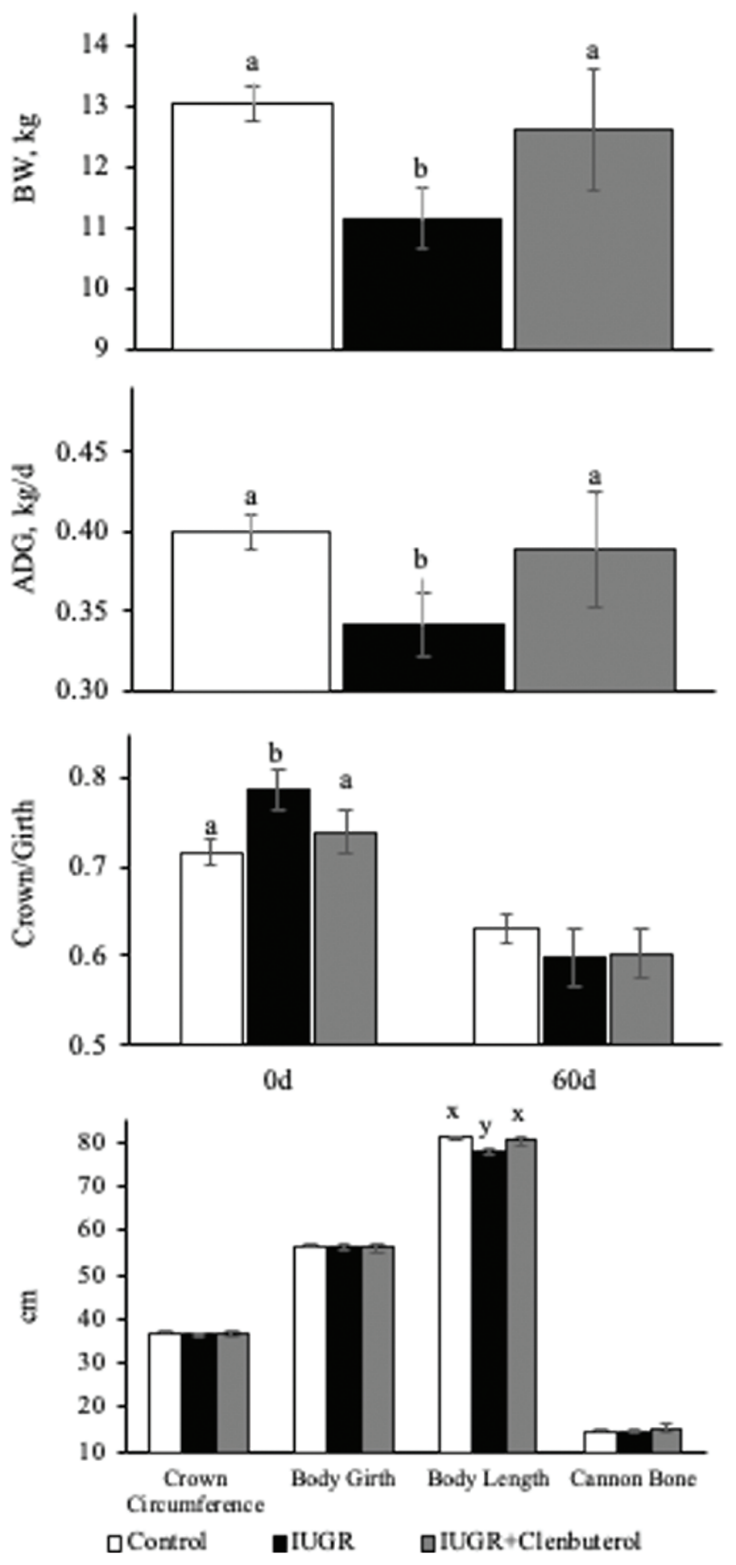
Growth metrics of birthweight (A), average daily gain (B), crown to girth ratios (C), and body length (D) in intrauterine growth restriction (IUGR)-born juvenile lambs supplemented daily with injectable clenbuterol. ^a,b^Means with different superscripts differ (*P* ≤ 0.05). ^x,y^Means with different superscripts tend to differ (*P* ≤ 0.10).

**Figure 2.**
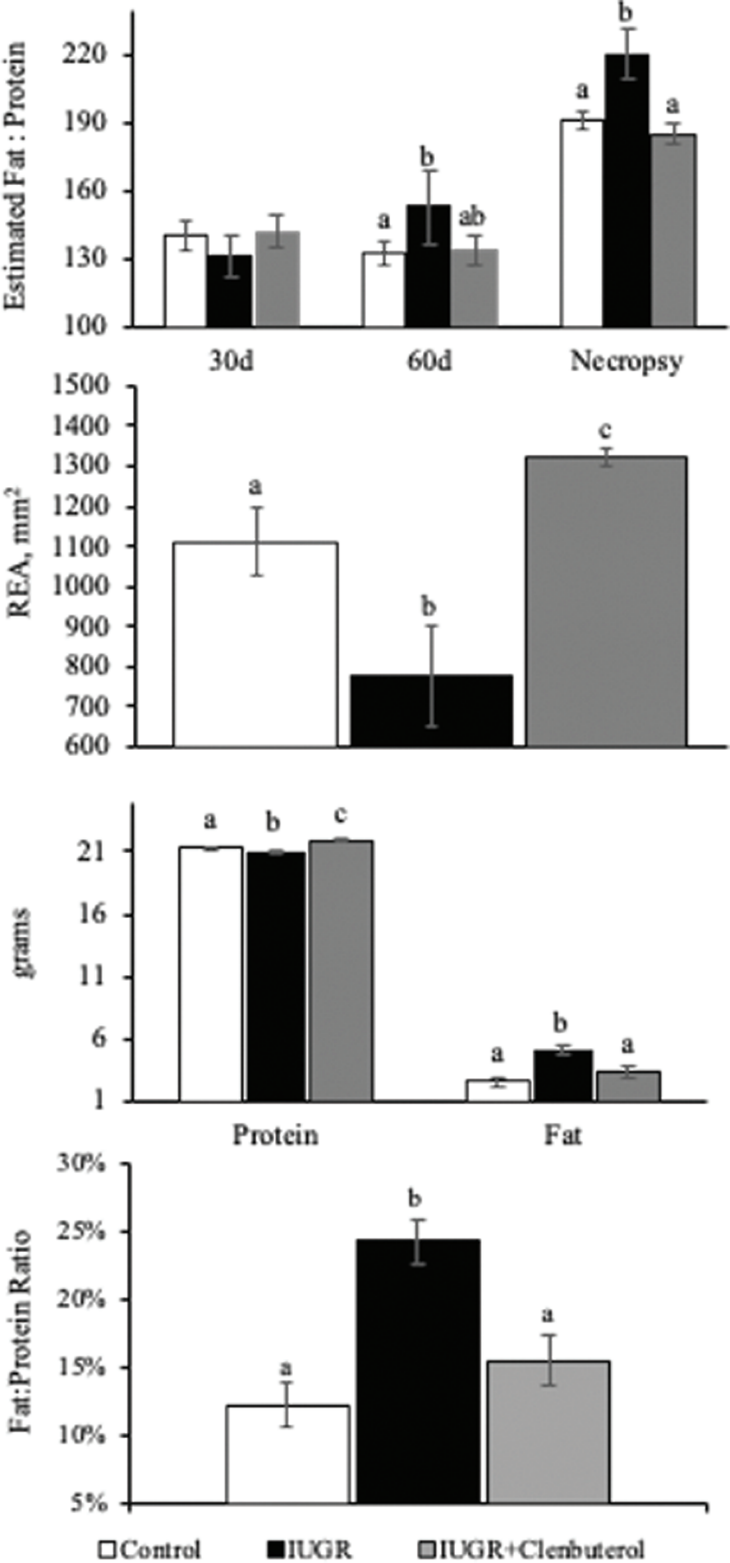
Muscle growth and composition metrics of BIA estimated fat to protein ratio (A), loin eye area (B), protein and fat content (C), and actual fat to protein ratio in intrauterine growth restriction (IUGR)-born juvenile lambs supplemented daily with injectable clenbuterol. ^a,b,c^Means with different superscripts differ (*P* ≤ 0.05).

Birthweights of IUGR lambs were 22% lighter (*P* < 0.05) than controls. Thirty-day and 60-d BW and average daily gains were less (*P* < 0.05) in unsupplemented IUGR lambs, but not in clenbuterol-supplemented lambs compared with controls. For all lambs, average daily gain from birth to 30 d tended to be greater (*P* = 0.10) than average daily gain from 30 to 60 d. Cranial crown circumference, body girth, crown/girth, and cannon bone length did not differ among groups. Crown-rump length was less (*P* ≤ 0.05) in unsupplemented IUGR lambs, but not in clenbuterol-supplemented IUGR lambs compared with controls (Figures 1 and 2).

At necropsy, hindlimbs and FDS muscles tended to be lighter (*P* ≤ 0.09) in unsupplemented IUGR lambs, but not in clenbuterol-supplemented IUGR lambs compared with controls. Hindlimb/BW and FDS/BW did not differ among groups. Heart weights tended to be lighter (*P* = 0.09) in unsupplemented and clenbuterol-supplemented IUGR lambs compared with controls, but heart/BW did not differ among groups. Lung weights and lung/BW were less (*P* ≤ 0.05) in unsupplemented IUGR lambs, but not in clenbuterol-supplemented IUGR lambs than in controls. Kidney weights were lighter (*P* ≤ 0.05) in unsupplemented IUGR lambs than controls, and were lighter (*P* ≤ 0.05) in clenbuterol-supplemented IUGR lambs than in unsupplemented lambs or controls. Brain and liver weights were not different among groups, but brain/BW tended to be greater (*P* = 0.07) for unsupplemented and clenbuterol-supplemented IUGR lambs than for control lambs. Loin-eye areas were smaller (*P* ≤ 0.05) in unsupplemented IUGR lambs but larger (*P* ≤ 0.05) in clenbuterol-supplemented IUGR lambs than controls.

At 60 d of age, fat-free lean mass was indicated by one of three equations to be lower (*P* ≤ 0.05) in unsupplemented IUGR lambs, but not in clenbuterol-supplemented IUGR lambs compared with controls (Table 1). Fat-free soft tissue mass did not differ. The estimated sum of the leg, sirloin, rack, shoulder, neck, riblets, shank, and lean trim mass (SUM); the sum of the leg, sirloin, loin, rack, and shoulder mass (LSRLS); and the sum of the leg, sirloin, and loin mass (LSL) were all less (*P* ≤ 0.05) in unsupplemented IUGR lambs, but not in clenbuterol-supplemented IUGR lambs than in controls. Nutrient composition estimations indicated no difference in moisture, lean mass, or protein content but less (*P* ≤ 0.05) fat content for unsupplemented IUGR lambs, but not in clenbuterol-supplemented IUGR lambs than for controls. Estimated crude fat content was indicated by one of three equations to be reduced (*P* ≤ 0.05) in unsupplemented IUGR lambs, but not in clenbuterol-supplemented IUGR lambs compared with control. In the carcass, estimated fat-free lean mass, fat-free soft tissue mass, SUM, LSLRS, LSL, protein, fat, and protein/fat were reduced (*P* ≤ 0.05) in unsupplemented IUGR lambs, but not in clenbuterol-supplemented IUGR lambs compared with controls. Proximate analysis of the loin muscle showed no differences in moisture, ash, or carbohydrate content, but loin fat content was increased (*P* ≤ 0.05) and protein content and protein/fat were reduced (*P* ≤ 0.05) in unsupplemented IUGR lambs compared with controls. In clenbuterol-supplemented IUGR lambs, loin fat content and protein/fat did not differ from controls but loin protein content was greater (*P* ≤ 0.05) than in controls.

**Table 1.** Live-animal body composition and carcass composition estimated by bioelectrical impedance in intrauterine growth restricted (IUGR) lambs supplemented daily with clenbuterol

Variables	Control	IUGR	IUGR + clenbuterol	*P-*value
60 d of age				
Fat-free lean mass, kg	13.38 ± 0.97	10.09 ± 2.87	13.34 ± 2.19	NS
Fat-free soft tissue, kg	13.88 ± 0.88	10.74 ± 2.70	13.73 ± 2.04	NS
Crude fat, g	210.5 ± 9.5^a^	183.7 ± 16.4^b^	214.6 ± 25.2^ab^	<0.001
SUM, kg	10.19 ± 0.34^a^	8.46 ± 0.5^b^	9.97 ± 1.30^ab^	<0.001
LSLRS, kg	7.1 ± 0.24^a^	5.96 ± 0.57^b^	6.95 ± 0.9^ab^	<0.001
LSL, kg	4.0 ± 0.17^a^	3.22 ± 0.42^b^	3.91 ± 0.59^ab^	<0.001
Moisture, kg	13.48 ± 0.69	11.55 ± 1.28	13.88 ± 1.8	NS
Protein, kg	4.09 ± 0.23	3.56 ± 0.40	4.32 ± 0.61	NS
Fat, kg	3.77 ± 0.30^a^	2.88 ± 0.56^b^	3.89 ± 0.8^ab^	< 0.001
Lean, kg	16.01 ± 0.83	14.15 ± 1.47	16.93 ± 2.23	NS
Necropsy				
Fat-free lean mass, kg	18.50 ± 0.49^a^	15.50 ± 0.55^b^	18.68 ± 1.34^a^	<0.001
Fat-free soft tissue, kg	21.68 ± 0.48^a^	18.73 ± 0.58^b^	21.77 ± 1.66^a^	<0.001
Crude fat, g	160.2.5 ± 3.0^a^	137.2 ± 9.5^b^	165.7 ± 17.6^a^	<0.001
SUM, kg	23.59 ± 0.54^a^	21.09 ± 0.74^b^	23.19 ± 2.31^ab^	<0.001
LSLRS, kg	15.13 ± 0.37^a^	13.44 ± 0.58^b^	14.86 ± 1.51^ab^	<0.001
LSL, kg	9.34 ± 0.22^a^	8.34 ± 0.34^b^	9.18 ± 0.91^ab^	<0.001
Moisture, kg	10.47 ± 0.27^a^	8.75 ± 0.46^b^	10.92 ± 1.29^a^	<0.001
Protein, kg	3.37 ± 0.10^a^	2.73 ± 0.16^b^	3.60 ± 0.45^a^	<0.001
Fat, kg	2.27 ± 0.11^a^	1.54 ± 0.19^b^	2.41 ± 0.55^a^	<0.001
Lean, kg	13.71 ± 0.39^a^	11.31 ± 0.58^b^	14.63 ± 1.66^a^	<0.001

SUM, sum of the leg, sirloin, rack, shoulder, neck, riblets, shank, and lean trim mass; LSLRS, sum of the leg, sirloin, loin, rack, and shoulder mass; LSL, sum of the leg, sirloin, and loin mass; NS, not significant.

^a,b^Means with differing superscripts differ (*P* < 0.05).

## DISCUSSION

In this study, we found that poor growth and body composition previously observed in fetal and neonatal IUGR lambs (Yates et al., 2016; Cadaret et al., 2019; Posont et al., 2019) persisted in juvenile-aged IUGR lambs. However, daily treatment with injectable clenbuterol for 2 mo improved postnatal growth and body composition in IUGR lambs. Morphometrics and lean tissue indicators in juvenile IUGR lambs reflected asymmetrical growth patterns that were compounded by deficits in feed efficiency, weight gain, and most of all muscling. This shows that adipose-driven catch-up growth expected in low-birthweight offspring (Yates et al., 2018) had not fully occurred in our IUGR lambs over this timeframe. Additionally, reduced gain for all lambs in the second month compared with the first month indicates decreasing capacity for growth over time. For IUGR offspring, this mean less opportunity to recover growth deficits. The impact of IUGR on muscle growth was particularly evident postmortem and shows the persistent effect of nutrient-repartitioning fetal adaptions. Loin-eye area and estimated lean mass were diminished in IUGR lambs, presumably the product of reduced fetal myoblast function (Yates et al., 2014) and impaired protein anabolism (Soto et al., 2017) that was not reconciled at the juvenile stage. Persistent poor muscle growth at this stage explains reduced yield and carcass merit previously observed at harvest (Greenwood et al., 2000). Although IUGR-born juvenile lambs had less total fat mass, the percentage of their loin muscle and soft tissue that was comprised of fat was increased. Moreover, estimated and actual protein-to-fat ratios were reduced in IUGR lambs even at this age, which perhaps reflects the beginning of the adipose-driven pattern of postnatal catch-up growth that arises from the reduced capacity for muscle growth (Cianfarani et al., 1999). Nevertheless, postnatal supplementation of clenbuterol recovered indicators of muscle-centric growth, thus improving a hallmark pathology of IUGR. This was perhaps unsurprising, as it reflects the β2 agonist’s ability to enhance muscle growth (Johnson et al., 2014). We conclude that the impact of IUGR on growth, particularly in muscle, extends beyond early life and continues in juvenile-aged offspring. However, daily treatment with clenbuterol demonstrated a potential avenue to recover muscle growth and weight gain in IUGR-induced low-birthweight livestock.

## IMPLICATIONS

Lambs born with low birthweight due to IUGR continued to exhibit diminished muscling, feed efficiency, and carcass merit as juveniles, which confirms that IUGR pathologies persist beyond early life. It also demonstrates the importance of developing preventative and therapeutic strategies to improve muscle growth and metabolic efficiency in low birthweight livestock. Our findings in clenbuterol-treated lambs demonstrates that postnatal pharmaceutical supplements may be a valid approach to improving diminished growth and body composition in IUGR offspring and thus warrant continued research.
